# The network collapse in multiple sclerosis: An overview of novel concepts to address disease dynamics

**DOI:** 10.1016/j.nicl.2022.103108

**Published:** 2022-07-14

**Authors:** Menno M. Schoonheim, Tommy A.A. Broeders, Jeroen J.G. Geurts

**Affiliations:** Department of Anatomy and Neurosciences, MS Center Amsterdam, Amsterdam Neuroscience, Amsterdam UMC, Vrije Universiteit Amsterdam, Amsterdam, The Netherlands

**Keywords:** Multiple sclerosis, Network, Connectivity, Hub, Efficiency, Cognition

## Abstract

Multiple sclerosis is a neuroinflammatory and neurodegenerative disorder of the central nervous system that can be considered a network disorder. In MS, lesional pathology continuously disconnects structural pathways in the brain, forming a disconnection syndrome. Complex functional network changes then occur that are poorly understood but closely follow clinical status. Studying these structural and functional network changes has been and remains crucial to further decipher complex symptoms like cognitive impairment and physical disability. Recent insights especially implicate the importance of monitoring network hubs in MS, like the thalamus and default-mode network which seem especially hit hard. Such network insights in MS have led to the hypothesis that as the network continues to become disconnected and dysfunctional, exceeding a certain threshold of network efficiency loss leads to a “network collapse”. After this collapse, crucial network hubs become rigid and overloaded, and at the same time a faster neurodegeneration and accelerated clinical (and cognitive) progression can be seen. As network neuroscience has evolved, the MS field can now move towards a clearer classification of the network collapse itself and specific milestone events leading up to it. Such an updated network-focused conceptual framework of MS could directly impact clinical decision making as well as the design of network-tailored rehabilitation strategies. This review therefore provides an overview of recent network concepts that have enhanced our understanding of clinical progression in MS, especially focusing on cognition, as well as new concepts that will likely move the field forward in the near future.

## Highlights

• Multiple sclerosis (MS) can be considered as a network disorder.
• This review discusses network concepts in order to understand progression in MS.
• Damage is hypothesized to lead to a “network collapse” and clinical progression.
• New concepts are discussed that will likely influence the field in the near future.
• These include brain wiring, how regions communicate and robustness to damage.

## 1 Introduction

Multiple sclerosis (MS) is a neuroinflammatory and neurodegenerative disease of the central nervous system. MRI measures of lesional damage are vital for the diagnosis of MS, but their relation with clinical presentation is limited, which is known as the “clinico-radiological paradox” (Barkhof, 2002, Benedict et al., 2004). As lesions continue to demyelinate axonal pathways, neuronal connections are disrupted and disconnected regions become atrophic (Azevedo et al., 2018). The observation that measures like atrophy are much more closely related to clinical performance has led to the development of more advanced quantifications of structural and functional changes in MS. Indeed, atrophy of strongly connected regions of the brain (i.e. hubs), like the thalamus, are most strongly related to clinical progression, which emphasizes the importance of network concepts in MS (Azevedo et al., 2018, Colato et al., 2021, Eshaghi et al., 2018). Over the course of MS, this continued structural disconnection induces changes to how brain regions communicate (Fleischer et al., 2019b, Jandric et al., 2022, Schoonheim et al., 2015b).

Importantly, these connectivity changes and network hub atrophy coincide with the development of cognitive impairment (Schoonheim et al., 2015b). Cognitive impairment has a profound effect on activities daily living and is central to MS disease burden over the entire course of the disease (Benedict et al., 2020). As the clinico-radiological paradox was especially apparent for cognition, many MS studies started focusing on functional connectivity (i.e. how much do brain regions communicate). These studies have shown a complex pattern of network changes in MS related to cognition but also fatigue (Manjaly et al., 2019) and disability (Faivre et al., 2016). Conceptually, these studies remain challenging given contradictory findings and heterogeneous methodologies. As such, the field now needs a clearer conceptual approach that truly captures MS as a network disorder, i.e. a conceptual framework that describes how focal (lesional) pathology might lead to global network changes and thus complex symptomatology (Chard et al., 2021, Fleischer et al., 2019b). A central hypothesis in recent years involves the notion that specific patterns of structural disconnection leads to less efficient wiring of the network, and after a critical threshold of efficiency loss has passed the network “collapses”, encompassing a state change with accelerated clinical progression (Schoonheim et al., 2015b).

This review functions as an overview of network concepts in MS, specifically focusing on those that were shown to be relevant for cognitive decline, since cognition has been studied most extensively and could function as an example for other disease dynamics in MS. In addition, new concepts are explored as well that are likely to become relevant in the future. Such concepts could help explain how network disconnection could lead to complex symptoms like cognitive impairment in MS, shedding further light on what might be going wrong in this complicated and multifactorial network collapse of the MS brain.

## 2 Network organization: A loss of efficiency in MS?

Before discussing individual findings in MS, it is important to first explain core concepts of the network neuroscientific field. The brain is a complex system and its functioning is dependent on structural and functional connections between many local as well as distant brain regions. In this framework, structural connections describe the anatomical links (i.e. edges) between brain regions (i.e. nodes) and represent the main routes through which brain regions can communicate (Bassett and Sporns, 2017, Venkadesh and Van Horn, 2021). Functional connections, on the other hand, characterize the synchronized activity between brain regions, i.e. the presumed strength of communication over the structural pathways (Bullmore and Sporns, 2009). As vastly different complex systems and networks feature shared organizational principles, these properties can be quantified and compared across networks. This field of “network neuroscience” presents a conceptual and mathematical framework (i.e. graph theory) to study this complex network (dis)organization in the MS brain (Bassett and Sporns, 2017, Blanken et al., 2021, Bullmore and Sporns, 2009).

A healthy brain, like most networks, shows a combination of high local clustering (i.e. segregated processing) due to extensive local cross-connectivity, as well as short average path-lengths between distant brain regions (i.e. integrated processing) due to a certain number of “shortcut” connections (Bassett and Bullmore, 2017). This combination is important for an efficient local and global network architecture (Latora and Marchiori, 2001) and was originally based on the “small world” principle postulated more than two decades ago (Watts and Strogatz, 1998). However, developments in the last twenty years since this conceptualization of a network was proposed have extended this conceptual field even further by describing *how* information is integrated. For instance, by focusing on the few crucial regions (i.e. connector hubs) that link distinct communities of brain regions (modules or subnetworks, such as the visual or motor systems), we can gain novel information on critical network structures where damage could have a drastic impact (Bassett and Bullmore, 2017, Power et al., 2013, van den Heuvel and Sporns, 2013). In recent years, the concepts of integration and segregation are still commonly used to describe “network efficiency”.

### 2.1 Network integration and segregation in MS

Measures of network integration capture how easily brain regions can communicate, based on the notion that shorter paths between brain regions result in network shortcuts allowing a faster distribution of information. In network neuroscience this concept can be quantified with measures such as the characteristic path length and global efficiency (Rubinov and Sporns, 2010). On the other hand, measures of network segregation characterize the capacity for specific processing within densely interconnected groups of brain regions (i.e. subnetworks) and can be quantified using measures such as clustering, local efficiency and modularity (Rubinov and Sporns, 2010).

In MS the disruption of the structural network has been analyzed in a few studies all showing reduced integration and segregation, particularly in cognitively impaired (CI) patients (Charalambous et al., 2019, Hawkins et al., 2020, Llufriu et al., 2017, Rimkus et al., 2019, Shu et al., 2016). This network change is also likely further exacerbated by a seemingly worse damage to long-range anatomical links, which were disproportionately affected compared to short-range links, and this was worst in CI compared to cognitively preserved (CP) patients (Lopez-Soley et al., 2020, Meijer et al., 2020). While most studies looking at segregation and integration look at the entire brain network, this can also be applied to subnetworks. For instance, a recent MS study showed that using an efficiency-based concept within the sensorimotor network (and thus the quantification of network efficiency loss herein) provided additional useful information to explain the severity of disability compared to only quantifying mean damage within this structural network (Pardini et al., 2015). Such network efficiency changes can already be seen in early stages, such as clinically isolated syndrome (CIS) (Shu et al., 2016). There are also some observations of increased structural network clustering and modularity (i.e. segregated processing), but only in such early stages (Fleischer et al., 2017, Fleischer et al., 2019b, Koubiyr et al., 2019, Tur et al., 2018, Tur et al., 2020). These changes have been interpreted as a (probably finite) compensatory structural phenomenon, which remains controversial. Overall, most studies showed a more segregated and less integrated structural network, particularly in patients with cognitive impairment (Fleischer et al., 2017, Fleischer et al., 2019a, Koubiyr et al., 2019, Koubiyr et al., 2021, Rimkus et al., 2019, Tur et al., 2020, Welton et al., 2020).

Functional networks in MS, however, seem to react in a much more complex way, and the link between network efficiency and cognition has been less clear. Some studies have suggested increased segregation in CI patients through an increased local efficiency (Schoonheim et al., 2013, Strik et al., 2021, Welton et al., 2020), also indicated by observations of increased modularity (Gamboa et al., 2014). However, reduced or unaltered segregation has been observed in the functional network of MS patients as well (Liu et al., 2017, Rocca et al., 2016, Schoonheim et al., 2012, Shu et al., 2016). Part of this discrepancy might lie in the different modalities used (e.g. functional MRI versus magnetoencephalography) and the ways of quantifying “network efficiency”. The latter highlights the importance of accurate semantics, as the way of quantifying “efficiency” has continuously evolved over time and the term is commonly used outside of the small-world framework as well. Apart from methodological considerations, there are also indications that some network changes are disease-stage specific, such as an initial increase and later decrease in connectivity, all related to clinical worsening (Faivre et al., 2016). As such, while the notion of an efficiency loss (as defined by the small-world framework) in MS populations has been useful, network concepts needed to evolve further to allow for the quantification of more complex network changes at specific stages.

Emerging concept 1: Hierarchical network organization Subnetworks of the brain are hierarchically organized and can be defined across multiple spatial scales (Meunier et al., 2010, Sporns, 2013). Therefore, the spatial characterization of network organization and “efficiency” in MS might be improved using multilevel subnetwork definitions (Akiki and Abdallah, 2019, Fan et al., 2019, Wang et al., 2021). Future studies should take this into account, which could lead to a better understanding and resolve some of the discrepancies related to the local network changes in MS patients with cognitive impairment.

### 2.2 Highly connected regions: Brain hubs in MS

Global network integration is supported by the high interconnectedness of specific network hubs (van den Heuvel and Sporns, 2011). These hubs can be identified using measures that capture how strongly a node is connected to other nodes (e.g. centrality) (van den Heuvel and Sporns, 2013). In structural networks, few changes were observed in the organization (e.g. ordering) of brain hubs of early MS patients (Koubiyr et al., 2019) and a loss of connections between hubs only seems to become more prominent in later stages (Shu et al., 2018).

In contrast, a disrupted hub organization of the functional network was already observed within 6 months after disease onset, with more hub disruption actually relating to better cognitive performance (Koubiyr et al., 2020). This finding of a beneficial change in network topology is actually quite rare in MS literature and seemingly limited to early stages only, with later disease stages mostly showing maladaptive network change (Rocca et al., 2010, Rocca et al., 2012, Rocca et al., 2016, Schoonheim et al., 2015b). This suggests that early functional rerouting might function as a compensatory mechanism to preserve the communication between distant brain regions (i.e. network integration), and is perhaps only possible when structural damage is not yet severe.

Although longitudinal studies on hub measures are still rare, recent observations again indicate disease-stage specific processes. More explicitly, centrality of the ventral attention network (VAN, also known as the salience network) has been observed to initially increase during the earliest stages before transitioning towards cognitive impairment (Huiskamp et al., 2021). This network change subsequently transfers towards a more central default-mode network (DMN) and thalamus in patients that show more severe cognitive impairment (Dekker et al., 2021, Eijlers et al., 2017, Rocca et al., 2016, Schoonheim et al., 2014). The DMN consists of many crucial hub regions in the brain (Kabbara et al., 2017), while the thalamus is a well-known integrative hub as well (Hwang et al., 2017), suggesting that an overload of hub-regions might play a central role in the development of cognitive impairment.

### 2.3 The tri-partite network axis in MS: An impaired dance between hubs

Based on recent data on the healthy brain, these aforementioned structures are known to be crucial for directing cognitive network information flow during specific situations. For instance, when focusing on specific cognitively charged content, the fronto-parietal network (FPN, also known as the central executive network, CEN) is actively processing information (Uddin et al., 2019). During this state, the VAN is actively monitoring queues and predicting what needs attention next (Uddin, 2015), aided by vigilance monitoring and information integration by the thalamus (Harrison et al., 2021, Hwang et al., 2017). However, when this active focus on particular content is no longer required, the VAN will aid in the suppression of cognitive networks like the FPN, and the emergence of the DMN as the dominant network during internally orientated processes (Davey et al., 2016, Uddin, 2015). Recent observations have further deepened our understanding of the role of this tri-partite network, indicating that the DMN is continuously shifting in and out of dominance during an active state as well. The DMN seems to hold a clear role for continuously linking new external information to previously acquired internal data (Yeshurun et al., 2021), which seems to be facilitated by specific FPN connections (Dixon et al., 2018), possibly even involving the cerebellum (Buckner, 2013).

As such, a continuously dominant FPN (Jandric et al., 2021) and DMN (Meijer et al., 2017) in MS at rest could indicate that this intricate network shifting might have become impaired, which is also supported by observations of a DMN bleed-through during active task processing (Rocca et al., 2014). In addition, recent findings within regions related to the DMN, such as altered thalamic (d'Ambrosio et al., 2017, Hidalgo de la Cruz et al., 2021a, Schoonheim et al., 2015a, Tona et al., 2014) and cerebellar connectivity (Sbardella et al., 2017, Schoonheim et al., 2021) could also indicate a maladaptive network change that no longer integrates all relevant information flow.

Emerging concept 2: Diversely connected regions Recent work has suggested that highly-connected hub regions might actually be less strongly involved in top-down active integration of information, but are rather important for integration by receiving information from multiple sources (i.e. subnetworks or modules) (Bertolero et al., 2017, Betzel et al., 2016). The participation coefficient is a measure that might actually reflect such an integration across multiple modules as it quantifies how connections of a node are distributed across (or “participate in”) different modules (Bertolero et al., 2017, Rubinov and Sporns, 2010). Initial work has shown altered cross-module participation of nodes in structural and functional modules at the clinical onset of MS (Koubiyr et al., 2019). Modularity, the way a network is separated into such modules, still requires more work in later disease stages of MS, where it remains understudied. It is likely that the observed severe disconnection of long-range connections at that disease stage should negatively affect the topology and functional diversity of such modules, and hence the participation coefficient (Betzel and Bassett, 2018, Meijer et al., 2020).

These recent innovations would not have been possible by only focusing on a constrained formal terminology of “network efficiency”, thus enlarging the network-based conceptual framework to include subnetworks and hubs is crucial for our understanding of progression in MS. In addition, the loss of interplay between crucially symbiotic subnetworks will obviously have a dire impact on the efficiency of such a system to adapt to environmental challenges (or tasks). As such, as concepts evolve, perhaps so should terminology. Thus, we believe the network collapse is more accurately described by a hub overload, resulting in an impaired interplay between subnetworks. Additionally, this hub overload may have an effect on the interaction between the structural and functional networks or the dynamic adaptability of the network, which we will explore in the following two sections.

## 3 Structure-function relationships in MS: Are functional and structural network changes interdependent?

The relatively fixed pattern of structural connections and the overall topology of the structural network shapes and constrains the overlaying functional network (Honey et al., 2010, van den Heuvel et al., 2009). The previous section described how the organization of either structural or functional networks are altered in MS, but are these changes interrelated? Can we gain additional insight into clinical symptoms in MS by specifically looking at the interplay between these two modalities?

### 3.1 Structure-function coupling

In the healthy brain, functional communication patterns can actually be predicted to a large degree by the pattern of anatomical links, i.e. structural connectivity corresponds strongly to functional connectivity (Honey et al., 2009). This similarity between structural and functional connectivity can be quantified by calculating the correlation between the two types of networks for each connection, i.e. structure–function coupling. Such coupling of direct connections was found to be increased during the first stages of MS, and greater coupling was related to worse cognitive performance (Koubiyr et al., 2021, van Dam et al., 2021). This suggests that the functional network becomes more strongly constrained by the structural network in cognitively impaired patients. This increased coupling could therefore indicate that communication between brain regions shifts from indirect to direct structural pathways, or that direct connections become less dynamic in their pattern of connectivity. As indirect structural connections play a significant role in shaping functional connectivity beyond these direct connections (Honey et al., 2009), future studies are now needed to specifically assess changes to direct and indirect connections with structure–function coupling measures, for instance using graph analytical approaches like network communicability (Li et al., 2013). In addition, as mentioned previously, there are clear indications of disease stage-specific effects, hence structure–function coupling should also be investigated in later stages of MS.

Emerging concept 3: Multilayer networks By connecting both network modalities in a so-called multi-layer network, both connectivity matrices are left unchanged, but actually are connected to form a “network of networks”, i.e. a more advanced way of quantifying structure–function relations within each node rather within individual edges. A relatively simple representation of a multilayer network is a so-called “multiplex” featuring one connection between structure and function within each node which might even be a constant, but this concept can be expanded to connections across layers and nodes to increase complexity. Recent work on the healthy brain has shown that emergent properties within such a multilayer relates to cognitive performance (Breedt et al., 2021), while such information was not captured by looking at the individual network layers separately (Crofts et al., 2016, Lim et al., 2019). In addition, such analyses have revealed an interdependent structure–function organization that could support the retention of brain functioning despite structural damage (i.e. robustness) which may be highly relevant for cognitive impairment in MS (Lim et al., 2019).

Emerging concept 4: Network control theory Looking at coupling and multilayer network topology allows us to study structure–function relations, but does not technically provide information on how a specific structural topology might drive and control certain functional network transitions. A new field within network neuroscience called “control theory” looks at such features in the structural brain network that determine how brain function dynamically evolves (Gu et al., 2015, Medaglia et al., 2017). In addition, network control theory would allow for a determination of how (for instance MS-related) structural damage affects the amount of energy that is needed to dynamically alter brain function and also to investigate which regions are most important for this change (Betzel et al., 2016). Previous research on the healthy brain has already suggested that regions involved in long-distance communication are important for functional network dynamics, particularly for moving to energetically distant states (Tang et al., 2020), which might therefore be further hampered by MS. Therefore, network control theory could provide important new insights into the observed alteration in functional network dynamics in MS.

## 4 Time-varying networks: Are brain dynamics altered in MS?

While most methods actually look at brain function as a summary measure over a certain window of around ten minutes, also known as “static” connectivity (Lurie et al., 2020), the brain is actually a highly dynamic and constantly evolving system, changing at much shorter timeframes (Bassett et al., 2011). Recent advances in methodologies now allow for the quantification for such a dynamic or time-varying connectivity. In the healthy brain, it has been observed that connectivity fluctuates even in the absence of an explicit task (i.e. resting-state) which means that static models may be overly simplistic (Bassett and Sporns, 2017, Lurie et al., 2020, Preti et al., 2017). In fact, it was observed that connectivity fluctuations themselves relate to cognition in healthy individuals, hence providing unique added information (Preti et al., 2017). What can we learn about cognitive impairment in MS by viewing the brain as this flexible, continuously adapting system?

### 4.1 Network variability in MS

To quantify how dynamically the network is fluctuating, most studies have looked at the variability of a network measure of interest, such as FC (e.g. (Bommarito et al., 2021, Leonardi et al., 2013, Lin et al., 2018)) or advanced measures (e.g. (Zhou et al., 2016)). One recent study has observed that the centrality of brain regions in the DMN, FPN and visual network were reduced in patients with MS and cognitive impairment (Eijlers et al., 2019). In this work, it was shown that the visual system and DMN normally are anti-correlated, which was lost in CI-MS. In addition to the observation of decreased hub dynamics, increased non-hub dynamics were also observed (e.g. salience, hippocampal and cerebellar (Bosma et al., 2018, Schoonheim et al., 2021, van Geest et al., 2018).

These observations indicate that hubs of MS patients with cognitive impairment become more rigid or gets “stuck” in a continuous hyperconnected state. In theory, this might be explained by the increased rerouting through hub regions in cognitively impaired patients, the so-called “hub overload”, as more incoming information could make these regions less adaptable, i.e. their increasing workload actually reduces their flexibility. Previous work has indicated that such hub regions in the so-called “rich club” form a stable core within the network, while connections with non-hub regions are usually much more unstable and dynamic (Bertolero et al., 2017, Gollo et al., 2015). As such, on their own, these regions often only act in a limited and slow fashion and their behavior usually reflects those shared by the entire network. As a result of MS, these hub-nonhub connections might become less fluid and adaptable, thereby making the network as a whole more rigid. This was also reflected in an analysis of static FC, showing a specific increase of hub FC with non-hub regions, but a stable core of hub-hub connections (Meijer et al., 2017).

Despite these few studies, the field of dynamic connectivity in MS is still very new. Future studies should look at the variability of other graph metrics to increase our understanding of how the brain might become more rigid across spatial scales. It is to be expected, for example, that global integration becomes less dynamic, but what happens on a smaller spatial scale?

### 4.2 Brain states in MS: Stable patterns in a dynamic environment

Similar to the transition from static towards dynamic approaches, the field has since evolved even further. The concept of “brain states” aims to study stable re-occurring patterns of FC that can be found within a particular timeframe. As it turns out, several time-varying connectivity parameters are hierarchically organized in the normal connectome (Lurie et al., 2020, Vidaurre et al., 2017). Even in the absence of an explicit task the functional network transitions between distinct states in a non-random order (Vidaurre et al., 2017, Zalesky et al., 2014), which are identified by observing recurrent network conformations (Miller et al., 2016). This new way of analyzing an order of events within a functional scan could reveal how the functional network dynamically moves between and behaves within states, which might be essential for understanding dynamic network integration and hence cognition in MS. Previous work has indicated that while indeed hub-nonhub connections are especially dynamic, there are also windows of increased whole-network efficiency that repeatedly occur, which enhance information processing in those timeframes (Zalesky et al., 2014).

In MS, an increase in switches between states was observed after two years of disease onset and this inversely related to lesion volume, but the relation to cognitive impairment was unexplored (Rocca et al., 2020). Another study has explored this relation and has shown that MS patients with cognitive impairment actually switched less between brain states compared to preserved patients, with particularly less time spend in a highly connected state, which could reflect aforementioned states of high network efficiency (d'Ambrosio et al., 2020). This reduced switching was also observed in a third study, where this dynamic network change was related to worse disability and CI, showing worst changes in progressive MS (Hidalgo de la Cruz et al., 2021b). The most recent study implementing this technique observed that even in an MS cohort with minor disability, those with more severe disease severity spent more time in a state of high connectivity, where subjects normally do not linger. Such dynamic network changes were related to depressive, fatigue and motor symptoms (Romanello et al., 2022).

**Fig. 1:**
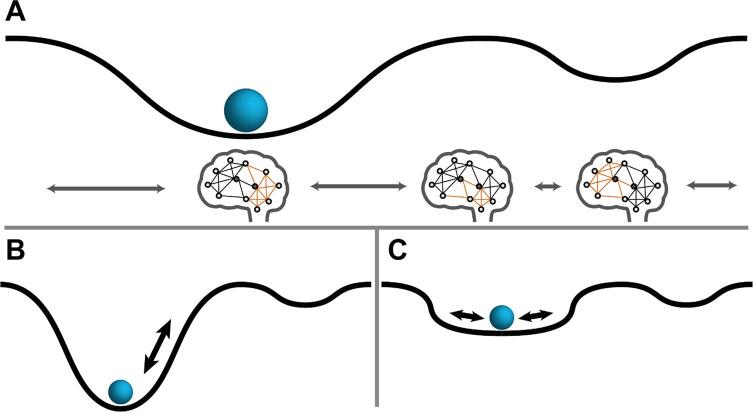
State changes as a function of network organization The “ball-and-cup” heuristic might help with thinking about how the functional network dynamically changes its organization. (A) The normal state, transitioning between all brain states continuously. All possible network conformations are represented by the surface, with the current organization of the network represented by the position of the ball (i.e. the functional network at a particular time-point). The ball can move over the surface (reflecting network dynamics), but this is governed by the nature of the surface. For example, the ball is more likely to remain in the cup, so the bottom of a cup represents frequently occurring network conformations (i.e. states). Still, after passing a critical threshold (i.e. moving over the hill), the ball moves into a new state. Different factors could affect how the ball moves within the landscape. (B) State resilience. The depth of a cup or the height of the hill affects how easily it can move between states. (C) State stability. The slope of the cup’s edges affects the movement within a state.

Possibly, these patients therefore enter highly integrative brain states less frequently, which would drastically impact their proficiency for continuous information processing. A major challenge in this field remains the actual deconvolution of these states in terms of what they represent. In general, resting-state scans do not allow us to fully deduct what each brain state represents in terms a corresponding “active-state”, hence studies combining the brain-state framework within resting-state and tasks or naturalistic stimuli could be important here (Shine and Poldrack, 2018, Sonkusare et al., 2019). Furthermore, a more in-depth exploration of how these switches between states are altered could yield even more information (e.g. using the “ball-and-cup” heuristic; see Fig. 1). Preliminary observations in MS seem to indicate deeper valleys for some states, while others have less steep edges, leading to less time spent in some, but more in others.

Emerging concept 5: Dynamic network reconfiguration In order to investigate how networks dynamically integrate information across subnetworks, the concept of brain states can be deepened further. For instance, by investigating the time-dependent organization of subnetworks and quantifying how this organization evolves over time (Bassett et al., 2011, Sporns, 2013). Brain regions are reconfigured across subnetworks more dynamically to integrate information across subnetworks (Li et al., 2019). Still, besides temporally flexible peripheral regions capable of such integration, a temporally stable core is likely very important as well, as mentioned previously (Bassett et al., 2013). Thus, characterizing how each “network subcompartment” is altered in cognitively impaired MS patients could give us complementary insights into the time-varying characteristics of network disintegration and efficiency loss. Accordingly, in one recent longitudinal study in MS, a destabilization of subnetworks seemed prominent in cognitively impaired MS patients, which worsened in cognitively declining patients (Broeders et al., 2022).

## 5 Longitudinal network changes: Can we predict cognitive decline in MS?

In this complex field of multiple network concepts and many variables, how can we differentiate between adaptive and maladaptive network alterations? Additionally, given the dynamic nature of network reorganization in specific disease stages, can we use network measures to predict cognitive decline at all?

Emerging concept 6: Network robustness The concept of network robustness can extend the abovementioned framework of computational models by assessing until when the topology of a network is maintained when a fraction of nodes or edges are removed (i.e. due to lesions) (Bullmore and Sporns, 2012). Robustness can be indirectly quantified using measures that generally reflect a network’s vulnerability to node removal, such as assortativity or curvature (Farooq et al., 2019, Rubinov and Sporns, 2010). Alternatively, robustness can be quantified by virtually inducing lesions to the structural network (Aerts et al., 2016, Gollo et al., 2018). Preliminary observations of reduced robustness in CI-MS networks (Farooq et al., 2020) could explain effects of individual buffer capacity or “cognitive reserve” as frequently observed in MS (Fuchs et al., 2019, Sumowski et al., 2014) and warrants future study. This approach could open new avenues of research into the collapse by combining empirical data with sophisticated computational models.

### 5.1 Adaptive or maladaptive network changes

Longitudinal studies are fundamental to advance our understanding of the network collapse and to be able to differentiate between adaptive (i.e. compensatory) or maladaptive network changes at different disease stages (Fleischer et al., 2019b, Jandric et al., 2022, Schoonheim et al., 2015b). In general, the assumption has been that network changes observed early in the disease that are not accompanied by cognitive worsening, reflect compensatory changes. However, it is important to note that such an assumption is mostly a “lack of overt maladaptation”, which is not the same. As such, future longitudinal studies are required to explicitly look at whether such changes actually relate to better clinical performance, before hopefully moving towards using such measures as a treatment target to stimulate with techniques like transcranial magnetic stimulation. Thankfully, there are promising indications that functional reorganization might play an important compensatory role at the early phase of the disease to counter the effects of structural damage (Fleischer et al., 2019b, Koubiyr et al., 2020), but more work needs to be done to fully understand when and why adaptive changes become maladaptive and which networks play an important role (Faivre et al., 2016). While the scarcity of longitudinal data of larger sample sizes and longer time-intervals remains a crucial problem in the MS field, computational models have recently evolved to such an extent that they can be used to simulate longitudinal functional network evolution based on structural input (Sanz Leon et al., 2013). Such models in MS have implicated that functional increases might actually be a direct result of structural disconnection (Patel et al., 2018, Tewarie et al., 2018).

### 5.2 Redefining and predicting the network collapse

**Fig. 2:**
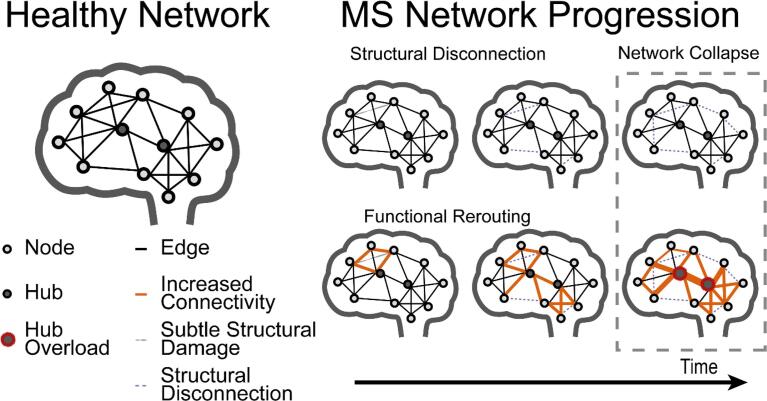
The network collapse as a cause for clinical progression and especially cognitive impairment in MS In early stages of MS, even subtle structural damage can induce extensive functional changes, usually in the form of hyperconnectivity. As structural damage becomes more severe and spreads, structural disconnection becomes apparent. Increased functional connectivity centers around hub regions, overloading these crucial network structures, leading to inefficient and rigid networks. The combination of exceeding certain thresholds of structural disconnection and hub overload is then thought to induce a “network collapse”, after which clinical progression accelerates.

As the field of connectomics in MS has grown exponentially in the past decade (Chard et al., 2021), the concept of a network collapse underlying clinical progression has been discussed frequently. What is still lacking, however, is a clear definition of what the collapse actually entails and which milestone events specifically herald its onset, and a systematic evaluation of how such a conceptual framework is clinically useful. Based on abovementioned conceptual innovations, we would propose the entire cascade of such a network collapse to be driven by a progressive loss of key structural connections in the brain network, exceeding a crucial threshold of structural network efficiency loss due to an impaired segregation and integration balance, leading to abovementioned complex functional patterns focused on the concept of hubs that become rigid and overloaded (Fig. 2).

More specifically, we hypothesize that this structural network segregation and disintegration drives functional network hubs to become overloaded as functional information is re-routed towards network hubs. This overload leaves hub regions, such as those encompassing the DMN and FPN, to lose their normal coordinated network dynamics that is crucial for cognition, possibly driven by an exhaustion of the controlling action of the salience network. This combination of structural disconnection and functional (and hence metabolic) exhaustion would start with the thalamus and then proceed towards the cortex, leaving cortical areas especially sensitive for a faster neurodegeneration in progressive MS. However, most of such findings have been cross-sectional and require longitudinal validation, as well as confirmation in other cohorts.

Apart from mapping the collapse itself, longitudinal data is also required to evaluate which network changes are most predictive for the collapse and hence clinical progression. Empirical longitudinal functional data showed that measures of functional connectivity (Hidalgo de la Cruz et al., 2021a) and functional network complexity (Nauta et al., 2021) are predictive of subsequent clinical decline, even when correcting for volumetric measures of structural damage in MS. These predictive variables were not necessarily the same as those found to be abnormal cross-sectionally in CI-MS (Nauta et al., 2021), indicating that the trajectory towards CI might not hold the same network mechanisms as those apparent after developing CI. Hence, it is important that future studies disentangle such predictive and “post-conversion” factors (Huiskamp et al., 2021).

## 6 Conclusion

Recent years have allowed us to better comprehend what the hypothesized network collapse in MS might represent. Firstly, the structural network seems to become more segregated and disintegrated, even though functionally the global integration of information seems largely preserved. This might be explained by a reorganization of the functional network, as communication in the functional network of these patients more often occurs through highly-connected and hence overloaded network hubs. This hub overload might leave the functional network more rigid and less adaptable, disrupting the key interplay between default-mode and fronto-parietal systems. While evolving concepts have led to these key discoveries, it is now essential to further evaluate structure–function relationships and push the methodological constraints for time-varying connectivity to further enhance our understanding of the network collapse. These future insights could then enable a clear definition and prediction of specific milestone (network) events as patients progress towards the collapse, using longitudinal observations and computational models, which could help clinical decision making as well as network-tailored rehabilitation strategies.

## CRediT authorship contribution statement

**M.M. Schoonheim:** Conceptualization, Investigation, Writing – original draft, Writing – review & editing, Resources. **T.A.A. Broeders:** Conceptualization, Data curation, Investigation, Software, Visualization, Writing – original draft, Writing – review & editing. **J.J.G. Geurts:** Conceptualization, Writing – original draft, Writing – review & editing, Supervision.

## Declaration of Competing Interest

The authors declare that they have no known competing financial interests or personal relationships that could have appeared to influence the work reported in this paper.
